# Mitochondria-Derived Damage-Associated Molecular Patterns in Neurodegeneration

**DOI:** 10.3389/fimmu.2017.00508

**Published:** 2017-04-26

**Authors:** Heather M. Wilkins, Ian W. Weidling, Yan Ji, Russell H. Swerdlow

**Affiliations:** ^1^Department of Neurology, University of Kansas Medical Center, Kansas City, KS, USA; ^2^University of Kansas Alzheimer’s Disease Center, Kansas City, KS, USA; ^3^Department of Molecular and Integrative Physiology, University of Kansas Medical Center, Kansas City, KS, USA; ^4^Department of Biochemistry and Molecular Biology, University of Kansas Medical Center, Kansas City, KS, USA

**Keywords:** damage-associated molecular pattern, mitochondria, neuroinflammation, neurodegeneration, sterile inflammation

## Abstract

Inflammation is increasingly implicated in neurodegenerative disease pathology. As no acquired pathogen appears to drive this inflammation, the question of what does remains. Recent advances indicate damage-associated molecular pattern (DAMP) molecules, which are released by injured and dying cells, can cause specific inflammatory cascades. Inflammation, therefore, can be endogenously induced. Mitochondrial components induce inflammatory responses in several pathological conditions. Due to evidence such as this, a number of mitochondrial components, including mitochondrial DNA, have been labeled as DAMP molecules. In this review, we consider the contributions of mitochondrial-derived DAMPs to inflammation observed in neurodegenerative diseases.

## Introduction

Inflammatory pathways are activated through either pathogen-initiated or damage-initiated events. Pathogen-associated molecular patterns (PAMPs) and damage-associated molecular patterns (DAMPs) activate similar inflammatory cascades and are therefore difficult to distinguish. Inflammation stimulated by DAMPs is an area of study that has recently gained notice. In particular, DAMPs derived from mitochondrial components are interesting due to the prokaryotic origin of this organelle. Furthermore, mitochondrial-derived DAMP molecules may play a role in heart disease, arthritis, liver disease, trauma, and sepsis (1–5).

Neuroinflammation and mitochondrial dysfunction are observed across numerous neurodegenerative diseases (6–8). Mitochondrial dysfunction can induce inflammation and *vice versa*. Mitochondrial dysfunction may modulate the release of mitochondria-derived DAMP molecules (9, 10). Here, we discuss the relationship between neuroinflammation, mitochondrial dysfunction, and mitochondria-derived DAMP molecules in the context of neurodegenerative diseases.

## Neuroinflammation in Neurodegenerative Diseases

Neuroinflammation is classically defined as proliferation and activation of microglia (microgliosis), and/or astrocytes (astrogliosis). Microglia are macrophage cells of mesenchymal origin, which monitor the central nervous system (CNS) for pathogens. Astrocytes are derived from the ectoderm and have numerous functions including metabolic support for neurons, regulating synapses, brain structure, and repair. Further evidence of neuroinflammation can encompass activation of inflammatory pathways, increased expression of cytokines or chemokines, and in some cases disruption of the blood–brain barrier (BBB) accompanied by infiltration of peripheral immune cells (such as T cells). Neurodegenerative diseases including Alzheimer’s disease (AD), Parkinson’s disease (PD), and amyotrophic lateral sclerosis (ALS) all have evidence of neuroinflammation pathology.

### Alzheimer’s Disease

Initial interest in neuroinflammation as a causative factor in AD centered on the link between reduced risk for AD and long-term non-steroidal anti-inflammatory drug (NSAID) use. Early evidence from epidemiological studies suggested that long-term use of NSAIDs led to a decreased risk of developing AD (11, 12). Subsequent studies found a correlation between apolipoprotein E (APOE, a genetic risk factor for AD) genotype and the protective effects gained from NSAID use. The correlation between decreased AD risk and NSAID use is greatest in individuals who harbor an APOE ε4 allele (13, 14). Furthermore, the age of the individual taking NSAIDs and the type of NSAID administered affect the association of AD risk reduction (15).

Clinical trials investigating whether NSAIDs might benefit AD subjects, though, were disappointing. Early trials experienced high attrition rates among participants due to adverse effects and thus did not provide clear answers (16, 17). A larger trial of one NSAID, tarenflurbil, showed largely negative results (18). However, a recent publication that showed positive memory and brain inflammation outcomes for a different NSAID, fenamate, in an AD mouse model has led to renewed interest (19).

Genome-wide association studies (GWAS) have also renewed interest for neuroinflammation as a potential causative factor for AD. In particular, a rare variant of triggering receptor expressed on myeloid cells 2 (TREM2), R47H, is associated with an increased risk for late onset Alzheimer’s disease (LOAD) (20). TREM2 is a membrane protein in myeloid cells (such as microglia), which modulates inflammatory pathways by inhibiting cytokine production. TREM2 is likely responsible for determining microglial phenotypes (i.e., M1 activation versus M2 activation). Furthermore, TREM2 is also important for lipid sensing, providing a further link between neuroinflammation and bioenergetic pathways (21). The R47H TREM2 variant leads to a reduction in microglial phagocytosis (22). Soluble TREM2 levels are increased in cerebral spinal fluid (CSF) of AD subjects, a parameter that positively correlates with gray matter volume but negatively correlates with diffusivity (sometimes considered a function of cell integrity) (23). Other single-nucleotide polymorphisms (SNPs) associated with AD risk also affect inflammatory pathways. These include SNPs in complement receptor 1, clusterin (CLU), major histocompatibility complex (MHC), class II, DR beta 5, ephrin type A receptor 1, inositol polyphosphate-5-phosphatase, and or Siglec-3 (24–29). The relationship of each of these genes to inflammatory signaling has been reviewed elsewhere (30).

Microglia and reactive astrocytes are closely associated with amyloid plaques in AD brain (31, 32). The quantity of interleukin-1 (IL-1) reactive microglia is increased sixfold in AD brain (33). Furthermore, microglial IL-1 expression correlates with plaque distribution (34). Levels of macrophage colony-stimulating factor, an activator of macrophages, was found to be increased in plasma and CSF from AD subjects compared to those with mild-cognitive impairment (35). A number of cytokines and chemokines, which can be released by macrophages or other immune cells, are increased in AD, including interleukin-1β (IL-1β), interleukin-6 (IL-6), tumor necrosis factor α (TNFα), interleukin-8 (IL-8), transforming growth factor β, and macrophage inflammatory protein-1α (36).

### Parkinson’s Disease

The discovery of reactive microglia in the substantia nigra of PD brain tissue provided early evidence for the role of neuroinflammation in PD (37). Further evidence for microglial activation in PD brain came from positron emission tomography (PET) imaging studies. PET studies showed increased microglial activation as evidenced by increased levels of peripheral benzodiazepine sites, a selective marker of activated microglia (38). Increased levels of glial cells expressing TNFα, IL-1β, and interferon-γ (IFN-γ) have also been observed in the substantia nigra of deceased PD subjects (39). Furthermore, microglial activation is observed in animal models of PD (36, 40).

Studies on astrogliosis in the PD brain have generated conflicting results. Early studies suggested increased astrogliosis in the PD brain. More recent studies have shown minimal levels of nigral astrogliosis, although astrocytic accumulation of α-synuclein was observed. The authors speculated that α-synuclein accumulation may cause astrocytes to be less reactive (41). A subsequent report showed minimal astrogliosis and an inverse correlation between levels of α-synuclein and astrogliosis in the PD brain, suggesting α-synuclein may suppress astrogliosis (42).

Similar to AD, PD risk is reduced with regular or chronic NSAID use. Long-term aspirin use is associated with less PD risk; however, non-aspirin-based NSAID use afforded better risk reduction. Furthermore, women who used aspirin in a chronic (greater than 24 months) or regular manner had a lower risk for PD than men (43).

Genome-wide association studies have identified the gene that encodes leucine-rich repeat kinase 2 (LRRK2) as a risk factor for sporadic PD. LRRK2 mutations are also associated with autosomal dominant forms of PD (44). Similar to TREM2 in AD, LRRK2 mediates microglial function (45, 46). LRRK2 facilitates vesicle trafficking and cytoskeletal remodeling and may skew microglia toward a pro-inflammatory phenotype (45–47).

Changes in peripheral inflammation may contribute to PD. Studies of peripheral inflammation showed an increased ratio of CD8+ T cells to CD4+ T cells in the blood of PD patients (48). Peripheral inflammation may affect the brain through disruption of the BBB, an event that is observed in PD subjects (49). Furthermore, peripheral inflammatory changes correlate with PD risk. Increased plasma IL-6 levels correlated with disease risk, although with a small sample size (50). CD8+ and CD4+ T cell reactivity is increased in the substantia nigra of PD patients. Furthermore, 1-methyl-4-phenyl-1,2,3,6-tetrahydropyridine (MPTP), a toxin used to generate a commonly utilized PD animal model, was found to increase T cell infiltration into the substantia nigra (51).

### Amyotrophic Lateral Sclerosis

Inflammatory changes have been observed in the CNS of ALS patients. Early studies in ALS spinal cord and motor cortex discovered accumulation of immunoglobulin G and complement (52). Subsequent studies found T cell infiltration and increased levels of MHC I and II antigens on macrophages and dendritic cells (53, 54). Immunohistochemical studies in postmortem brain tissue showed increased levels of phagocytic and leukocyte surface proteins on microglia, as well as evidence to support the infiltration of activated lymphocytes into the precentral gyrus (55). PET studies using PK11195, a ligand that binds microglia, have provided further evidence of microglial activation in ALS (56).

Astrogliosis is observed within the spinal cord (ventral and dorsal horns) and brain (cortical gray matter and subcortical white matter) in postmortem ALS subjects (57). Microgliosis is evident in the spinal cord ventral horn, the corticospinal tract, and the motor cortex (55). The role of microglia and astrocytes in ALS disease progression is further supported in transgenic mutant superoxide dismutase 1 (SOD1) ALS mouse models. Selective deletion of mutant SOD1 from microglia (which also removes expression from peripheral myeloid cells) slowed disease progression (58). Similar results were observed when mutant SOD1 expression was deleted from astrocytes (59). Conversely, deleting mutant SOD1 expression from motor neurons had no effect on disease progression or survival, but did delay disease onset (58). These studies emphasize the potential role of neuroinflammation in ALS disease progression.

Genome-wide association studies in ALS have linked inflammatory genes with disease outcome. A SNP in CX3C chemokine receptor 1 (CXCR31, fractalkine receptor) is associated with reduced survival in sporadic ALS subjects; however, this SNP fails to associate with ALS risk (60). CXCR31 is important for the migration of leukocytes and may play a role in microglial migration (61). RNA seq and ingenuity pathway analysis of postmortem ALS spinal cord samples found upregulation of inflammatory pathways, with TNFα being a predicted upstream regulator of inflammation in these ALS samples (62).

Neuroinflammation is evident in AD, PD, and ALS. However, whether or not neuroinflammation contributes to disease onset, progression, and risk requires further study. To this end, a growing appreciation of a relationship between mitochondrial dysfunction and inflammatory pathways may provide insight into this important question. Below, we discuss evidence for mitochondrial dysfunction in AD, PD, and ALS.

### Primary Mitochondrial Diseases

Primary mitochondrial diseases are caused by mutations in mitochondrial DNA (mtDNA) or nuclear DNA genes that encode mitochondrial proteins. Diseases caused by mtDNA mutations include Leber’s hereditary optic neuropathy, myoclonic epilepsy and ragged red fiber, neuropathy ataxia and retinitis pigmentosa, Kearns–Sayre syndrome, and Leigh’s syndrome. Diseases caused by a nuclear DNA mutation of which affects a mitochondrial localized protein include Friedreich’s ataxia, Wilson’s disease, and Mohr–Tranebjaerg syndrome. Commonly, these diseases manifest in neurological symptoms. A few of these diseases are associated with neuroinflammation pathology.

Friedreich’s ataxia is caused by autosomal recessive inheritance of a mutant *Frataxin* gene. The product of the *Frataxin* gene is responsible for iron homeostasis within mitochondria, and loss of this gene in Schwann cells leads to reduced mitochondrial respiration, inflammation, increased mitochondrial iron concentrations, and cell death (63–65). COX2 expression is elevated in both animal models and Friedreich’s ataxia patient lymphocytes, an indicator of increased inflammation (66).

For subjects with Leigh’s syndrome, mtDNA mutations occur in several genes including ATPase 6, ND 1–6 (NADH dehydrogenase), and CO3 [cytochrome oxidase (COX)] (67, 68). These subjects often have deficient ETC enzyme activities (67). In a mouse model of Leigh’s syndrome, evidence of neuroinflammation is abundant (69). However, inflammatory markers have not been measured from human subject tissues.

Wilson’s disease is caused by a mutation in the *ATP7B* (ATPase copper transporting β polypeptide) gene and is characterized by liver disease, ataxia, parkinsonism, seizures, and reduced cognition (70, 71). This gene encodes a copper transporting ATPase that localizes to mitochondria and affects mitochondrial copper levels (70, 72). Subjects with this mutation have reduced ETC function (73, 74). Pentraxin 3, a marker of inflammation, is elevated in the serum of Wilson’s disease subjects (75).

Despite the association of mitochondrial dysfunction and neuroinflammation or inflammation (discussed below), these processes have not been extensively studied in primary mitochondrial diseases. Future research endeavors into this area would likely benefit our understanding of these diseases.

## Mitochondrial Dysfunction in Neurodegenerative Diseases

The Krebs cycle and oxidative phosphorylation occur in the matrix and inner mitochondrial membrane, respectively. Oxidative phosphorylation requires the mitochondrial respiratory chain. These bioenergetic pathways generate the high energy compound adenosine triphosphate (ATP) (76). Mitochondria and bioenergetic intermediates generated within mitochondria regulate cell signaling pathways (including pro-inflammatory responses, as discussed below).

The brain comprises approximately 2% of the body’s weight yet consumes about 20% of its oxygen uptake. The brain requires high amounts of energy for numerous processes, including neurotransmitter production and synaptic activity. Therefore, the brain is highly susceptible to mitochondrial dysfunction, which has been observed in several neurodegenerative diseases including (but not limited to) AD, ALS, and PD (77–80). Furthermore, mitochondrial dysfunction declines with age, and age is the greatest risk factor for these neurodegenerative diseases (78, 80). Mitochondrial dysfunction can lead to increased reactive oxygen species (ROS) production, decreased ATP production, alterations in mitochondrial membrane potential, damage to mtDNA, and activation of cell death pathways (81).

### Alzheimer’s Disease

In postmortem AD brains, decreased COX function, reduced intact mitochondrial number, and increased mitochondrial autophagy have been reported (78, 82–86). Mitochondrial dysfunction appears to be systemic in AD, as deficits in COX activity are apparent in AD patient fibroblasts and platelets (83, 87–89). Changes in mtDNA may drive cell signaling changes, bioenergetic pathway deficits, and histopathological hallmarks of AD. Cytoplasmic hybrid (cybrid) studies in which mtDNA from human AD subjects is transferred into a donor cell line that lacks its own mtDNA provides a system in which mtDNA-derived biochemical and molecular consequences can be assessed. The cybrid model system controls for nuclear DNA alterations, as patient mtDNA is transferred into the context of a consistent nuclear DNA background (90). Cybrid cells generated using AD patient mtDNA have reduced COX activity, increased ROS production, and increased Aβ deposition (90, 91).

Evidence of mtDNA mutations, deletions, and oxidative modifications are present in AD subjects (92–97). mtDNA is inherited from the mother, and interestingly a maternal inheritance pattern for AD has been noted. This maternal inheritance pattern is associated with early changes in brain atrophy and mitochondrial biomarkers (98–103). Finally, mitochondrial haplotypes are associated with increased AD risk (104–106). These studies suggest changes in mitochondrial function, possibly at the level of mDNA maintenance and inheritance, are important in AD pathology.

### Parkinson’s Disease

The most studied respiratory chain aberration in PD is a deficit in complex I activity. Initial insight into this deficit stems from cases of recreational drug users exposed to MPTP. After MPTP exposure, individuals developed parkinsonian symptoms and at autopsy were found to have degeneration in the substantia nigra, similar to that observed with PD. This degeneration occurred in the absence of Lewy bodies (or aggregated α-synuclein). Following its accidental discovery, MPTP was adapted to produce monkey and rodent models of PD (107–109). MPTP is oxidized to MPP+, which accumulates in neurons and is a potent complex I inhibitor (107, 110).

Complex I deficits are observed in postmortem brain, platelets, and fibroblasts from PD subjects (111, 112). The observed deficits in complex I activity could be driven by oxidative damage to its catalytic subunits or altered mtDNA (90, 113, 114). Cybrid cell lines generated from PD subject mtDNA recapitulate the complex I deficit. Sporadic PD cybrid cell lines also show reduced mtDNA copy number, reduced ATP, cell death pathway activation, and a relatively depolarized mitochondrial membrane potential (113–115).

Parkinson’s disease risk is associated with mtDNA haplotype, similar to AD (116–118). Changes to mtDNA are also observed in PD, such as mutations and deletions (119–121). Polymorphisms of mtDNA polymerase γ influences PD risk as well (122). Overall, mitochondrial function and mtDNA inheritance and maintenance are important to the pathology of PD.

### Amyotrophic Lateral Sclerosis

ALS subjects have evidence of mitochondrial dysfunction within the CNS and periphery. CNS mitochondrial abnormalities include altered mitochondrial morphology, mitochondrial inclusions/aggregates, reductions in COX activity, and lower mtDNA levels with increased levels of mtDNA point mutations and deletions (123–128). COX deficits, lowered mitochondrial number, altered mitochondrial calcium levels, and mtDNA deletions, are also observed in ALS subject muscle (129–131). Lymphocytes from ALS subjects have reduced mitochondrial maximum respiration, liver mitochondria appear swollen, and platelet mitochondria manifest a depolarized mitochondrial membrane potential with increased apoptosis (132–135). Cybrid cells generated from ALS patient mtDNA have alterations in antioxidant enzyme activity and reduced complex I activity (136). The association of ALS with distinct mtDNA haplotypes is under investigation (137).

## Mitochondrial Dysfunction and Inflammation

Mitochondria are important modulators of innate immunity pathways. Mitochondria-derived ROS, calcium, and ATP are signaling molecules that activate inflammatory responses. Under conditions in which mitochondria are damaged, such as accumulated mtDNA mutations or mitochondrial dysfunction (possibly through aberrant ROS production), a sustained inflammatory response and downstream pathological inflammation could ensue. A relationship between mitochondrial dysfunction and inflammatory signaling is discussed below.

Mitochondria can directly activate inflammasome signaling. Mitochondria-derived ROS activate the NLR family pyrin domain containing 3 (NLRP3) inflammasome pathway. NLRP3 is normally associated with the endoplasmic reticulum membrane, but upon activation is redistributed to nuclear and mitochondrial membranes, where it oligomerizes with apoptosis-associated speck-like protein containing a CARD (ACS) and pro-caspase 1 to form the NLRP3 inflammasome (138–140). Mitochondria also mediate inflammatory pathway activation through redox sensitive proteins (140).

Mitochondrial dysfunction initiates inflammation across various models. *In vitro* and *in vivo* experiments demonstrate complex II inhibition using 3-nitropropionic acid (3NP) induces microglial activation and reduces the ability of microglia to undergo alternate activation (141). Human microglial cells treated with 3NP became activated and showed increased ROS production and cell death rates. Similar results were observed following intrastriatal 3NP injection in adult rats, where microglial activation, ROS production, and cell death were increased (142). Chronic subcutaneous injections of rotenone in rats (a complex I inhibitor that is used to model PD in rodents) increases IL-1β within the hypothalamus. These rats also displayed a decreased number of tyrosine hydroxylase positive neurons in the substantia nigra, perturbed locomotion, and sleep abnormalities (143).

Conversely, pro-inflammatory cytokines also modulate mitochondrial function. For example, TNFα can decrease complex I activity, reduce ATP production, depolarize the mitochondrial membrane potential, increase ROS, and lower activities of complexes II and IV (depending on the cell type). In hepatocytes, TNFα uncouples mitochondria and increases ROS production. This ROS production is generated from complex I and III of the respiratory chain and initiates NFĸB activation (144, 145). Similar effects have been reported in L929 cells (a mouse fibroblast cell line), a leukemia cell line, and 3T3-L1 adipocytes (146–148). In a mouse hippocampal cell line (HT22), TNFα induces mitochondrial respiration deficits and a loss of mitochondrial membrane potential (149). IL-1β also yields similar effects on mitochondrial function (150). Nitric oxide (NO), another inflammatory signaling molecule, disrupts mitochondrial membrane potential and inhibits COX activity. In human retinal pigment cells, TNFα, IL-1β, and IFN-γ increase the production of both mitochondrial- and NADPH oxidase-derived ROS (151).

*In vivo* studies also suggest pro-inflammatory molecules influence mitochondrial function. Intraperitoneal injection of lipopolysaccharide in rodents increases brain cytokine and inflammatory receptor expression [including IL-1β, toll like receptor 2 and 4, microglial scavenger receptor A (SRA), and Fc receptor (FCγRII)] in a region-specific manner. The authors also reported increased microglial number and mitochondrial functional alterations, including decreased glutathione (an antioxidant) and increased complex II/III activity (152).

A clear relationship between mitochondrial dysfunction and inflammatory cascades exists. Both of these pathological hallmarks are present across multiple neurodegenerative diseases. Mitochondria-derived DAMP molecules could provide a further link between these pathologies. Below, we discuss mitochondria-derived DAMP molecules, potential routes of release, and evidence for their role in neuroinflammation.

## Mitochondria-Derived DAMPs: Inducers of Neuroinflammation?

Previously identified mitochondria-derived DAMP molecules include mtDNA, cardiolipin, ATP, mitochondrial transcription factor A (TFAM), cytochrome *c*, and formyl-methionine-labeled peptides (30). Formyl-methionine-labeled peptides may also include mitochondrial protein-derived cryptides. Cryptides are endogenous, fragmented functional peptides. Pro-inflammatory cryptides from mtDNA and nuclear DNA-encoded mitochondrial proteins were recently described (153–155). As reviewed in Table 1, each of these molecules has been observed to initiate a pro-inflammatory phenotype under various disease and pathological states.

**Table 1:** **Mitochondria-derived damage-associated molecular pattern molecules**.

Molecule	Disease or pathologic context	Reference
Mitochondrial DNA	Trauma, heart failure, arthritis, Parkinson’s disease, Alzheimer’s disease, aging	(1, 3, 156–158)
Cardiolipin	Arthritis, bowl disease, myocardial infarct/heart disease	(159–166)
Adenosine triphosphate	Atherosclerosis, lung inflammation/fibrosis	(167, 168)
Formyl-methionine-labeled peptides	*In vitro* neuron degeneration, inflammatory activation of neutrophils and macrophages	(169–172)
Transcription factor A	*In vitro* microglial activation, hemorrhagic shock	(173, 174)
Cytochrome *c*	Arthritis, liver injury, myocardial infarct/heart disease, SIRS	(175, 176)

Damage-associated molecular pattern molecules activate inflammatory signaling in a manner similar to PAMPs. The danger molecule is recognized by a pattern-recognition receptor (PRR), and adaptor molecules initiate intracellular signaling cascades and cytokine production. Similar to PAMPs, mitochondria-derived DAMPs are recognized by various PRRs. These include TNF receptor, NLRP3, IL-1 receptor, nucleotide-binding oligomerization domain-like receptor, receptor for advanced glycation end products, formyl peptide receptors (FPR or FPRL1), and purogenic receptors. With the increasing recognition of mitochondria-derived DAMP molecules as pathogenic instigators in various diseases (Table 1), recent studies have begun to explore their contribution to neuroinflammation.

### Mitochondria-Derived DAMP Molecules in Neuroinflammation

In the periphery, TFAM functions as a potent DAMP molecule (Table 1). Recently, it was found that TFAM, in combination with IFN-γ, can activate human microglial cells, human peripheral blood monocytes, and THP1 monocytic cells (173). Cotreatment of THP1 monocytic cells with IFN-γ and TFAM or TFAM alone lead to an increase in cytokine expression (including IL-1β, IL-6, and IL-8). While treatment of SH-SY5Y neuroblastoma cells with IFN-γ and TFAM was not toxic, exposure to conditioned medium from monocytic cells activated with IFN-γ and TFAM-induced SH-SY5Y cell death. Finally, mitochondrial proteins extracted from THP1 monocytic cells produced effects similar to TFAM.

Degraded and oxidized mtDNA can initiate pro-inflammatory pathways in astrocytes. Exposure of mtDNA to hydrogen peroxide can induce its degradation, and these mtDNA degradation products are found in human CSF and plasma (9). Furthermore, transfection of mouse primary astrocytes with oxidant-initiated, degraded mitochondrial polynucleotides caused a pro-inflammatory response. This inflammatory astrocyte phenotype was characterized by the upregulation of IL-6, monocyte chemotactic protein 1, TNFα, and IL-1β (9). It was also previously reported that mtDNA is degraded in response to hydrogen peroxide in HA-1 hamster ovarian cells, an effect that was not observed with nuclear DNA or cytoplasmic RNA. Degradation of the mitochondrial genome was apparent in both mtDNA and mitochondrial RNA species (10). These observations suggest a mechanism for mtDNA degradation, and also for the downstream activation of glial cell pro-inflammatory phenotypes.

Mitochondrial components induce inflammation in microglial (BV2) and neuronal (SH-SY5Y) cells (177). In the defining experiments, these cells were exposed to mitochondrial lysates prepared from SH-SY5Y cells containing mtDNA or alternatively SH-SY5Y cells lacking mtDNA. BV2 microglial cells exposed to mitochondrial lysates containing mtDNA had increased TNFα, IL-8, and matrix metalloproteinase 8 mRNA but decreased TREM2 mRNA. Furthermore, NFĸB nuclear localization was increased. These effects were not observed when BV2 microglial cells were exposed to mitochondrial lysates prepared from cells that lacked mtDNA. In SH-SY5Y neuronal cells exposed to mitochondrial lysates containing mtDNA, TNFα mRNA and NFĸB protein expression were elevated. In addition, mitochondrial lysate-exposed SH-SY5Y cells showed increased amyloid precursor protein (APP) mRNA and protein. Changes in APP expression or pro-inflammatory pathways did not occur when SH-SY5Y cells were exposed to mitochondrial lysates that lacked mtDNA.

Mitochondria-derived DAMP molecules induce neuroinflammation *in vivo*. We recently observed stereotactic injection of mitochondrial lysates or mtDNA into rodent hippocampi induced pro-inflammatory changes (178). Mitochondrial lysates increased hippocampal TNFα mRNA and decreased TREM2 mRNA expression. In addition, NFĸB phosphorylation was elevated in the cortex, while glial fibrillary acidic protein (GFAP) protein levels were elevated within the hippocampus. Hippocampal mtDNA injection lead to increased hippocampal TNFα mRNA but reduced hippocampal TREM2 mRNA, increased GFAP hippocampal protein expression, elevated cortical NFĸB phosphorylation, increased cortical colony-stimulating factor 1 receptor protein expression, and increased levels of phosphorylated AKT within the cortex. Beyond these inflammatory changes, whole mitochondria lysates increased protein and mRNA levels of endogenous rodent APP and Aβ_1–42_. These effects on APP and Aβ_1–42_ were not observed following injection of mtDNA. Overall, these studies provide evidence that mitochondria-derived DAMP molecules are capable of inducing neuroinflammation, as well as altering AD-related pathways.

While some studies suggest Aβ is pro-inflammatory and acts as a DAMP molecule, a recent study interestingly suggests Aβ has antimicrobial properties (179). In models that overproduce Aβ, including rodent, cell culture, and worm models, the severity of fungal and microbial infections was reduced. Injection of the bacterium *S. typhimurium* into the brain of an AD mouse model (5XFAD model) increased the propensity of Aβ to form plaques, and Aβ colocalized with the bacteria. In addition, the 5XFAD mice injected with *S. typhimurium* showed increased survival and reduced meningitis. This study further found that oligomerization of Aβ was required for this antimicrobial property to manifest. Earlier *in vitro* studies also showed Aβ initiated antimicrobial activity against several bacterial and fungal microorganisms. Aβ inhibited bacterial growth, and brain homogenates from AD subjects (containing Aβ) were also capable of inhibiting microbial growth (180). These studies suggest a possible connection between mitochondria-derived DAMP molecules, neuroinflammation, APP metabolism, and Aβ.

### Mitochondria-Derived DAMP Molecules As Biomarkers of Brain Integrity

Cell-free circulating mitochondrial components (DAMPs) such as mtDNA are altered in trauma. For example, in children with traumatic brain injury (TBI), CSF mtDNA levels are elevated. CSF mtDNA levels also correlate with TBI outcome. In children who survived a TBI, CSF mtDNA levels were in the lower range, while in children whose outcome included either death or severe disability CSF mtDNA levels were in the upper range. CSF levels of another DAMP molecule, high mobility group box 1, correlated with mtDNA levels in these subjects. Overall, mtDNA appears to represent a potential CSF mitochondrial DAMP biomarker for TBI and to have the potential to correlate with patient outcomes (3).

Circulating mtDNA is also associated with aging. In a study of 831 Caucasian subjects spanning ages 1–104 years, plasma mtDNA levels began to increase after the fifth decade of life. Plasma mtDNA levels further correlated with cytokine levels, specifically TNFα, IL-6, and regulated on activation normal T cell expressed and secreted (RANTES). Subjects with the highest levels of plasma mtDNA also had the highest levels of these cytokines (TNFα, IL-6, and RANTES), while subjects with the lowest plasma mtDNA levels had the lowest amounts of measured cytokines (156).

Circulating mtDNA may also potentially have the ability to serve as an AD or PD biomarker. Cell-free CSF mtDNA levels are reduced in clinically asymptomatic subjects with a genetically defined increased AD risk and clinically symptomatic AD subjects (157). However, no differences were observed for subjects with frontotemporal lobar degeneration. In a separate study, PD subjects were found to have lower levels of cell-free CSF mtDNA (158). While some may argue lower cell-free CSF mtDNA negates the possibility of a mitochondria-derived DAMP-induced neuroinflammation, other evidence contradicts this idea. It is important to note levels of another AD biomarker, Aβ, are elevated in the brain but significantly reduced in CSF from AD subjects (181, 182). Therefore, it is difficult to draw conclusions regarding CSF biomarker data and causation without sufficient knowledge of the mechanisms that underlie those biomarker changes.

### Specific Release of Mitochondria-Derived DAMP Molecules within the CNS

An important question relevant to the issue of mitochondria-derived DAMPs is how might these intracellular molecules access the extracellular space? While cellular components are released during necrotic cell death, more specific process through which mitochondria are released to the brain’s extracellular compartment have recently been described. One of these processes has been termed transcellular mitophagy. Neurons are large cells and can span long distances. Mitochondria continuously move between the cell body and dendrites, and between the cell body and axons. When peripheral mitochondria cease to function properly, they were believed to return to the cell body for disposal through the process of mitochondrial autophagy or mitophagy. Data now indicate neurons can also export mitochondria to surrounding glial cells, where they then undergo mitophagy. This process was first described to occur within the optic nerve and the cortex (183). In a separate study, it was found that astrocytes also transfer mitochondria to neurons (184). The transfer of mitochondria from astrocytes to neurons enhanced neuronal survival following ischemia reperfusion injury and required signaling from cluster of differentiation 38, cyclic ADP ribose, and calcium.

## Conclusion

**Figure 1:**
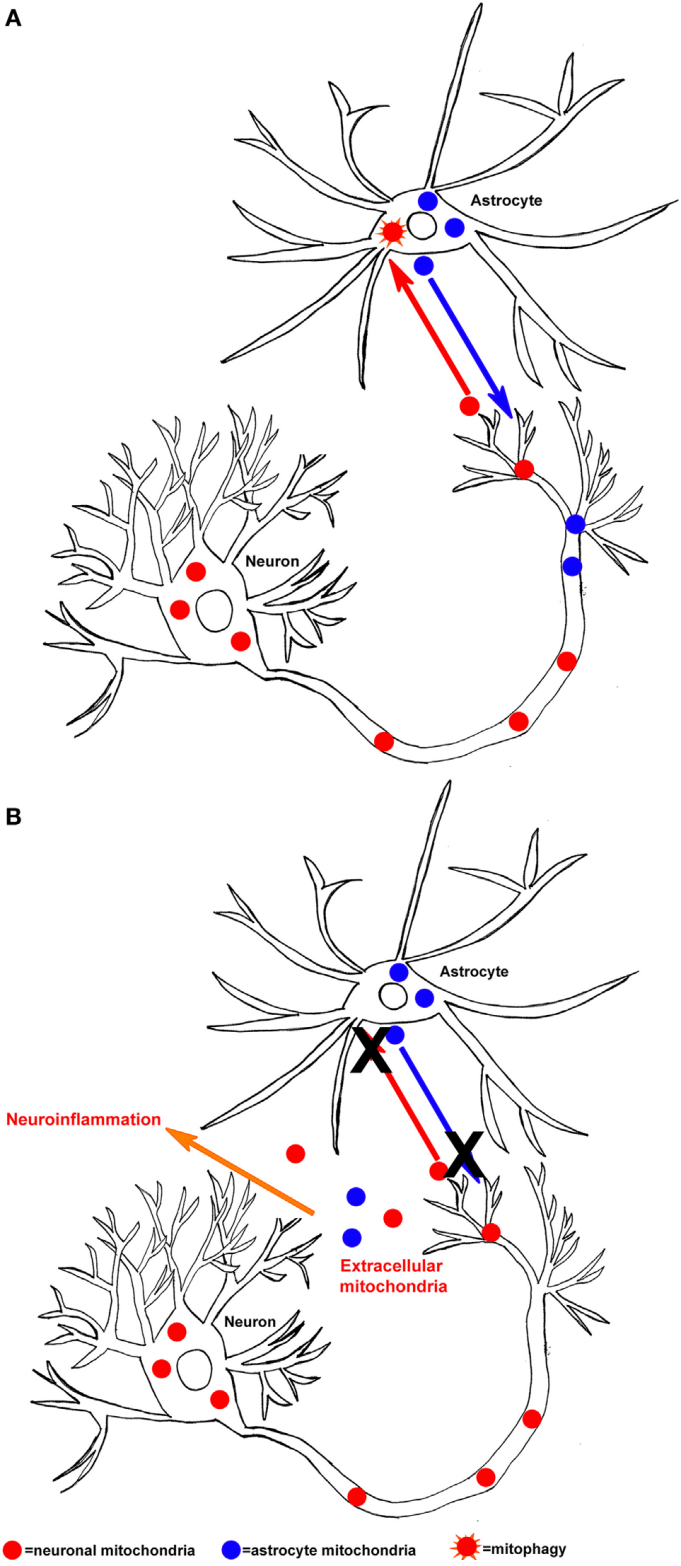
**Hypothesized mechanism of mitochondrial-derived damage-associated molecular pattern molecule released in the central nervous system**. **(A)** Neurons can release mitochondria (neuronal mitochondria are shown in red) to astrocytes, where they then undergo mitophagy (orange star shape). In addition, astrocytes can release mitochondria (astrocyte mitochondria are shown in blue) to neurons, under conditions of bioenergetic stress. **(B)** If the process of mitochondrial exchange between neurons and astrocytes malfunctions (X), then mitochondria and their components could be released into the extracellular space and initiate neuroinflammation.

In specific neurodegenerative diseases, if mitochondrial dysfunction overwhelmed the ability of neurons and astrocytes to adequately perform mitophagy, then mitochondria-derived DAMP molecules could predictably facilitate neuroinflammation (Figure 1). Clearly, a rational case can be made that mitochondria-derived DAMP molecules may contribute to neurodegenerative disease-associated neuroinflammation. However, important questions remain about how mitochondrial DAMPs contribute to neurodegenerative diseases and neurodegenerative disease-related pathologies. Future directions should focus on if and how specific mitochondrial-derived molecules initiate neuroinflammation. More specifically, how does mitochondrial dysfunction contribute to the release of DAMP molecules and is this process upstream or downstream of other disease pathologies? As this line of investigation moves forward, studies to address issues of cause versus consequence should help resolve these important knowledge gaps.

## Author Contributions

HW was responsible for writing the article. IW and YJ contributed to sections of the article. RS edited and provided direction for the article.

## Conflict of Interest Statement

The authors declare this review article was completed in the absence of any commercial or financial relationships that could be constructed as a potential conflict of interest.

## Abbreviations

3NP, 3-nitropropionic acid; ACS, apoptosis-associated speck-like protein containing a CARD; AD, Alzheimer’s disease; ALS, amyotrophic lateral sclerosis; APOE, apolipoprotein E; APP, amyloid precursor protein; ATP, adenosine triphosphate; BBB, blood–brain barrier; CD33, Siglec-3; CD38, cluster of differentiation 38; CLU, clusterin; CNS, central nervous system; COX, cytochrome oxidase; CR1, complement rector 1; CSF, cerebral spinal fluid; CSF1R, colony-stimulating factor 1 receptor; CXCR31, CX3C chemokine receptor 1 or fractalkine receptor; cybrid, cytoplasmic hybrid; DAMP, damage-associated molecular pattern; EPHA1, ephrin type A receptor 1; ER, endoplasmic reticulum; FCγRII, Fc receptor or CD32; FPR or FPRL1, formyl peptide receptors; FTLD, frontotemporal lobar degeneration; GFAP, glial fibrillary acidic protein; GWAS, genome-wide association study; HLA-DRB5/DRB1, major histocompatibility complex, class II, DR beta 5; HMGB1, high mobility group box 1; IFN-γ, interferon-γ; IgG, immunoglobulin G; IL-1, interleukin-1; IL-1β, interleukin-1β; IL-6, interleukin-6; IL-8, interleukin-8; INPPD5, inositol polyphosphate-5-phosphatase; IP, intraperitoneal; LPS, lipopolysaccharide; LRRK2, leucine-rich repeat kinase 2; MCI, mild-cognitive impairment; MCP-1, monocyte chemotactic protein 1; M-CSF, macrophage colony-stimulating factor; MHC, major histocompatibility complex; MIP-1α, macrophage inflammatory protein-1α; MMP-8, matrix metalloproteinase 8; MPTP, 1-methyl-4-phenyl-1,2,3,6-tetrahydropyridine; mtDNA, mitochondrial DNA; NLRP3, NLR family pyrin domain containing 3; NO, nitric oxide; NOD, nucleotide-binding oligomerization domain-like receptors; NSAID, non-steroidal anti-inflammatory drug; PAMP, pathogen-associated molecular pattern; PD, Parkinson’s disease; PET, positron emission tomography; PRR, pattern-recognition receptor; RAGE, receptor for advanced glycation end products; RANTES, regulated on activation, normal T cell expressed and secreted; ROS, reactive oxygen species; SNP, single-nucleotide polymorphism; SOD1, superoxide dismutase 1; SRA, microglial scavenger receptor A; TBI, traumatic brain injury; TFAM, transcription factor A; TGF-β, transforming growth factor β; TNFα, tumor necrosis factor α; TREM2, triggering receptor expressed on myeloid cells 2.
